# Transcriptomic analysis implicates the involvement of *RBM20* in Fuchs’ endothelial corneal dystrophy with *TCF4* repeat expansion

**DOI:** 10.1371/journal.pone.0332512

**Published:** 2025-09-17

**Authors:** Xunzhi Zhang, Ze Yu, Aundrea K. Westfall, Kunyi Han, V. Vinod Mootha, Chao Xing

**Affiliations:** 1 Eugene McDermott Center for Human Growth and Development, University of Texas Southwestern Medical Center, Dallas, Texas, United States of America; 2 Children’s Medical Center Research Institute, Dallas, Texas, United States of America; 3 Department of Statistics, University of Wisconsin, Madison, Wisconsin, United States of America; 4 Department of Ophthalmology, University of Texas Southwestern Medical Center, Dallas, Texas, United States of America; 5 Lyda Hill Department of Bioinformatics, University of Texas Southwestern Medical Center, Dallas, Texas, United States of America; 6 O’Donnell School of Public Health, University of Texas Southwestern Medical Center, Dallas, Texas, United States of America; University of Minnesota Medical School, UNITED STATES OF AMERICA

## Abstract

**Background:**

Late-onset Fuchs’ endothelial corneal dystrophy (FECD) is a degenerative disease of cornea manifesting during the fourth decade of life or later. An intronic trinucleotide repeat expansion (CTG18.1) in the transcription factor 4 (*TCF4*) gene is estimated to account for two thirds of FECD cases. There is a high degree of similarity between the transcriptomic profiles with (RE+) and without (RE-) the expansion. The molecular mechanisms of FECD and the difference between the two FECD types remain to be elucidated.

**Method:**

Analyses were based on publicly available RNA sequencing datasets of human corneal endothelial tissues. We compared the distributions of differentially expressed genes between the RE+ and RE- transcriptomic profiles for a given co-expression network module. Upstream regulator analysis, alternative splicing analysis, motif enrichment analysis, and structure prediction were conducted.

**Results:**

The expression levels of ribonucleic acid binding motif protein 20 (*RBM20*) were upregulated in both RE+ cases and RE+ controls. Consistently, its motif was enriched in the skipped exon events of RE+ subjects compared with RE- subjects. There were skipped exon events in three genes—*DST*, *FNBP1* and *SORBS1*— consistently identified in RE+ subjects out of the documented RBM20 target genes in the literature.

**Conclusion:**

RBM20 may represent an RE+ specific factor in the pathogenesis of FECD. The increase of *RBM20* expression in RE+ individuals may contribute to the disease by repressing the inclusion of exons.

## Introduction

Fuchs’ endothelial corneal dystrophy (FECD) is an age-related degenerative disease of cornea and the most common cause of corneal transplantation worldwide [1]. The hallmark of FECD is diffuse thickening of Descemet’s membrane with focal excrescences called guttae, along with the progressive loss of the normal morphology and cell density of the corneal endothelium. Impaired endothelial function causes corneal edema, leading to blurred vision and potential vision loss. FECD can be classified into early-onset and late-onset subtypes based on the age at which it becomes clinically apparent. The rare early-onset subtype generally appears within the first decade of life, whereas the more frequent late-onset form manifests during the fourth decade or later. The prevalence of the late-onset FECD was estimated to be > 4% in the population of European ancestry over the age of 40 and increased with age [2,3].

FECD is a multifactorial disease involving both genetic and environmental factors [4–6]. Variants in more than 10 genes/loci have been shown to be either causal or associated with the disease [7–15], among which the association between the expansion of a cytosine-thymine-guanine (CTG) trinucleotide repeat polymorphism (CTG18.1) in the intron of transcription factor 4 gene (*TCF4*) and FECD was replicated in many cohorts of different ethnicities [16–27]. The CTG18.1 repeat expansion is most prevalent in people of European ancestry and least in African ancestry, which is consistent with FECD prevalence [28]. It was determined to be causal for the disease [26] and estimated to explain two-thirds of cases [19]. There were several plausible pathogenic mechanisms proposed (for a review see, e.g., reference [29]); in particular, it was shown the expanded CUG repeats within the *TCF4* transcript bind and sequester RNA splicing factors such as MBNL1 and MBNL2, resulting in widespread splicing dysregulation [30–32]. Note that except for *DMPK* the remaining candidate genes do not harbor repeat expansions nor have obvious interconnections or shared common pathways.

Despite the different genetic causes, there is a striking similarity between the transcriptomic profiles of FECD patients with (RE+) and without (RE-) the CTG18.1 expansions in terms of both differential expression compared to controls and gene co-expression networks [33]. Multiple biological processes are significantly impacted in both RE+ and RE- FECD—mitochondrial functions, energy-related processes, ER-nucleus signaling pathway, demethylation, and RNA splicing are enriched with down-regulated genes, whereas small GTPase mediated signaling, actin-filament processes, extracellular matrix organization, stem cell differentiation, and neutrophil mediated immunity are enriched with up-regulated genes. It is of interest to note the RNA splicing process is negatively enriched in both types of FECD patients. Moreover, alternative splicing (AS) and gene expression changes in RE+ controls foreshadow the changes observed in late-stage RE+ cases [30]. Therefore, the mechanisms underlying FECD remain to be fully elucidated.

RNA splicing is a critical post-transcriptional modification during the expression of eukaryotic genes, removing the introns and joining the exons together [34]. The variety of the combinations in RNA splicing, i.e., AS, contributes to the diversity and complexity of transcriptome and proteome [35], with an estimate that ~95% human multi-exon genes are alternatively spliced [36]. Meanwhile, RNA mis-splicing underlies various human diseases [37]. Ribonucleic acid binding motif protein 20 (RBM20) is an AS regulator with two zinc finger domains, an RNA recognition motif (RRM), an arginine/serine-rich region, a leucine-rich region and a glutamate-rich region [38]. Localized inside the nucleus, RBM20 binds to a conserved UCUU RNA core element through its RRM domain [39–41]. It has been shown mutations in RBM20 causes RNA mis-splicing leading to dilated cardiomyopathy [42–44]; nevertheless, the involvement of this splicing factor in other diseases is unclear.

We previously performed a meta-analysis of FECD based on the published RNA-seq datasets of human corneal endothelial tissues [33], and revealed a great magnitude of similarity between RE+ and RE- transcriptomic profiles. In the current study we integrated the gene differential expression results and co-expression networks to identify RBM20 as an RE+ specific factor. AS and motif analyses were performed to corroborate the discovery.

## Methods

### RNA sequencing datasets and analyses

The RNA-seq datasets used in the study were listed in Table 1. We previously performed a transcriptomic meta-analysis of FECD based on bulk RNA-seq datasets of human corneal endothelial tissues [33]. There were a total of 25 RE+ cases, 8 RE- cases, and 19 RE- controls included in the meta-analysis from 3 studies—I: 6 RE+ cases, 4 RE- cases, and 9 RE- controls accessible at Gene Expression Omnibus (GEO; GSE142538) [30]; II: 8 RE+ cases, 4 RE- cases, and 6 RE- controls accessible at Sequence Read Archive (SRA; PRJNA524323 [45]; III: 11 RE+ cases and 4 RE- controls at GEO with accession number GSE112201 [46]. In addition, there were 6 RE+ controls—termed “pre-symptomatic”—from study I included in the current study. These RE+ control tissues were obtained from the eye bank of Transplant Services at UT Southwestern. They were from donors—mean age 46.8 years—with normal endothelial morphology examined by specular microscopy and without the CTG18.1 repeat expansion genotyped by short tandem repeat assay [30]. The differential expression meta-analysis of RE+ cases vs. RE- controls and RE- cases vs. RE- controls was previously described [33]. Genes with the false discovery rate (FDR) < 0.05 and consistent directional changes among individual studies were regarded as differentially expressed genes (DEGs). The same data processing procedure was followed to perform differential expression analysis of RE+ controls vs. RE- controls in dataset I.

**Table 1:** Bulk and single cell RNA sequencing datasets of human tissues.

Study	Phenotype	Genotype	Sample size	Sequencing type
I. GEO: GSE142538	case	RE+	6	bulk
		RE-	4	
	control	RE+	6	
		RE-	9	
II. SRA: PRJNA524323	case	RE+	8	bulk
		RE-	4	
	control	RE-	6	
III. GEO: GSE112201	case	RE+	11	bulk
	control	RE-	4	
IV. GEO: GSE155683	Unknown	Unknown	4	single cell

Phenotype is defined in terms of Fuchs’ endothelial corneal dystrophy affection status. Genotype RE+ and RE- refer to with and without the *TCF4* CTG18.1 expansion allele (≥40 repeats). The bulk and single cell RNA-seq experiments were performed using human corneal endothelium and cornea-conjunctival tissues, respectively. All bulk RNA-seq samples were sequenced with paired-end reads on an Illumina platform with read length of 150, 125, and 101 bp in I, II, and III, respectively. The single cell RNA-seq libraries were prepared using Chromium Single Cell 3′ Library & Gel Bead Kit (v3, 10x Genomics) and sequenced on an Illumina NovaSeq 6000.

We also queried a single-cell RNA sequencing (scRNA-seq) dataset of 4 adult cornea-conjunctival tissues accessible at GEO:GSE155683 [47]. Note it was not documented whether these donors had FECD, neither was their CTG18.1 repeat expansion genotype. The gene expression matrix and annotations of cell types were downloaded from http://retinalstemcellresearch.co.uk/CorneaCellAtlas/. Data visualization was realized using Seurat (v4.4.0) [48].

### Coupling gene differential expression profiles and co-expression networks

**Fig 1:**
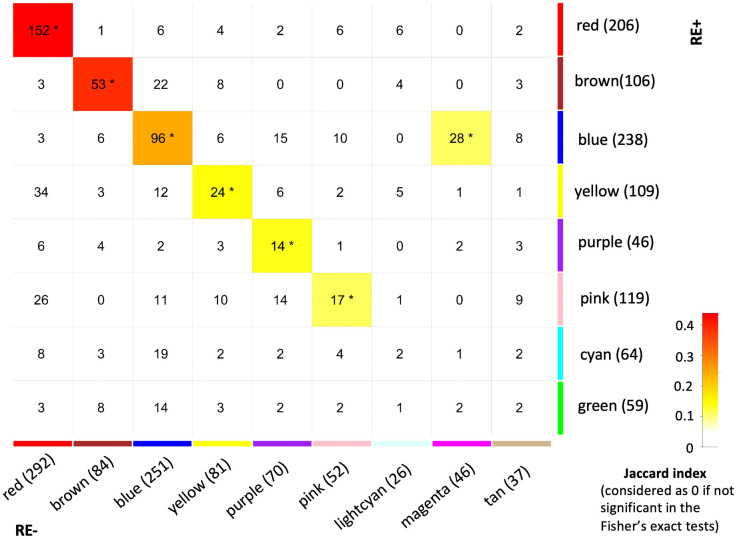
Correlation of gene co-expression networks in Fuchs’ endothelial corneal dystrophy patients with (RE+) and without (RE-) the *TCF4* CTG18.1 expansion. This matrix lists the pairwise comparison of genes in modules of RE+ and RE- co-expression networks [33]. The number of genes in each module was shown after the module name in the parenthesis. The number showed in each block is the count of intersected genes. * indicated module pairs with significant correlation (FDR < 0.05). The color indicated the Jaccard index, and was left blank (value = 0) if the correlation was not significant.

We previously constructed both RE+ and RE- cases gene co-expression networks and identified both similar and distinct modules based on the extent of overlapping genes [33]. The co-expression analysis identifies biological processes and functions orchestrated by genes with similar expression patterns [49]. A cluster of highly connected genes constitute a module, usually denoted by an assigned color code, which can be interpreted by functional enrichment analysis etc. There were 6 module pairs (red, brown, blue, yellow, purple, and pink) showing significant similarity in terms of constituted genes between RE+ and RE- profiles; in addition, the magenta module in RE- was also significantly correlated with the blue module in RE+ (Fig 1). For each pair of modules, we compared the distributions of DEGs between RE+ and RE-. First, all module genes were considered by performing Fisher’s exact test. Second, sensitivity analysis considering only the genes shared between a pair of modules was carried out by examining with McNemar’s test.

### Upstream Regulator Analysis

To identify the transcriptional regulators that can potentially explain the observed gene expression changes in a module, specifically, the RE+ brown module, we integrated the upstream regulator analysis (URA) and differential expression analysis results to prioritize the candidate upstream regulators. First, URA was performed based on the results of gene differential expression meta-analysis of RE+ cases vs. RE- controls using the QIAGEN Ingenuity Pathway Analysis (IPA) software [50]. It tests whether there is a statistically significant overlap between an input gene list and the genes that are regulated by a regulator using the one-sided Fisher’s exact test. Next, we filtered for regulators that met three criteria: (1) the regulator itself was differentially expressed in RE+ cases; (2) the regulator was not differentially expressed in RE- cases; and (3) the regulator had differentially expressed targets in the RE+ brown module. Further, we focused on regulators differentially expressed between the RE+ controls and RE- controls.

### Alternative splicing analysis

Differential AS analyses of RE+ cases vs. RE- controls, RE+ controls vs. RE- controls, and RE+ cases vs. RE- cases, were performed within each dataset using rMATS turbo (v4.1.2) [51]. Both junction and exon reads were used for counting. A differential splicing event is defined by meeting four criteria: (1) FDR < 0.05; (2) change of inclusion level > 5%; (3) the difference of average inclusion or skipping read counts between the two conditions > 5; and (4) the average inclusion read counts > 5. Sashimi plots were generated using rmats2sashimiplot (v2.0.4, https://github.com/Xinglab/rmats2sashimiplot). In the current study we examined the AS events in the 45 genes documented to be RBM20 targets in the literature (S1 Table) [42,44] and filtered for consistent events identified in RE+ cases vs. RE- controls of all three datasets.

### Motif analysis

RNA-binding protein (RBP) motif enrichment analysis for the splicing events was performed using rMAPS2 [52] based on the rMATS output. It maps the RBM20 motif (UCUU) to the regions with differential splicing events meeting criteria (1) and (2) in the **Alternative splicing analysis** section above and detects the significantly enriched regions (P < 0.05) compared to the background non-differential splicing regions. The analysis was performed for skipped exon (SE) and mutually exclusive exon (MXE) events, separately, which were reported to be the predominant AS types at the RBM20 targets [40].

The binding between the RRM domain of human RBM20 protein and the target RNA fragments of *FNBP1*, *SORBS1*, and *DST* with the UCUU motif was predicted using AlphaFold 3 [53]. The input RBM20 protein sequence consisted of residues 511−619 (UniProt:Q5T481), covering the RRM domain and its C-terminal helix region as previously reported [41]. The input RNA sequences were derived by first obtaining the coding strand DNA sequences of the target exons— chr9: 129,915,966−129,915,980 (hg38; exon 11), chr10: 95,351,209−95,351,376, (exon 25), and chr6: 56,468,982–56,468,999 (exon 98) for *FNBP1*, *SORBS1*, and *DST*, respectively— and their upstream and downstream fragments using bedtools getfasta (v2.27.1) [54] and then converting them to RNA sequences. The top-ranked predicated models were visualized with Mol* Viewer (https://molstar.org/) [55].

## Results

### Identification of RBM20 as an RE+ specific factor

Of the 7 co-expression module pairs with similar gene composition, the proportions of DEGs were significantly different between RE+ and RE- in 4 pairs—red-red, brown-brown, blue-blue, and blue-magenta (Table 2). Of interest, the proportions of DEGs were higher in RE+ than in RE- only between the brown-brown module pair, but lower between the other three pairs. This observation was consistent either considering all the genes in a module pair or considering only the shared genes between the pair. Given that the proportion of DEGs in the RE- brown module was the lowest (9 out of 84 genes) in all the 14 modules we hypothesize that the transcriptomic (dys)regulation of the brown module genes is RE+ FECD specific.

**Table 2:** Distribution of significantly differentially expressed genes between module pairs in Fuchs’ endothelial corneal dystrophy (FECD) co-expression networks with (RE+) and without (RE-) the *TCF4* CTG18.1 expansion.

Module pair	RE+		RE-		Proportion Difference	P-value
	Yes	No	Yes	No		
All module genes						
red – red	99	107	190	102	−0.17	$\text{1}.\text{5}\times {\text{10}}^{-\text{3}}$
**brown – brown**	**37**	**69**	**9**	**75**	**0.24**	$9.7\times 10^{-4}$
blue – blue	56	182	103	148	−0.18	$\text{3}.\text{3}\times {\text{10}}^{-\text{4}}$
yellow – yellow	50	59	22	59	0.19	$\text{7}.\text{1}\times {\text{10}}^{-\text{2}}$
purple – purple	22	24	37	33	−0.05	1.0
pink – pink	45	74	15	37	0.09	1.0
blue – magenta	56	182	23	23	−0.26	$\text{3}.\text{6}\times {\text{10}}^{-\text{3}}$
Shared module genes						
red – red	84	68	119	33	−0.23	$\text{8}.\text{3}\times {\text{10}}^{-\text{6}}$
**brown – brown**	**19**	**34**	**4**	**49**	**0.28**	$2.1\times 10^{-3}$
blue – blue	20	76	51	45	−0.32	$\text{2}.\text{0}\times {\text{10}}^{-\text{5}}$
yellow – yellow	9	15	3	21	0.25	$\text{5}.\text{4}\times {\text{10}}^{-\text{1}}$
purple – purple	5	9	7	7	−0.14	1.0
pink – pink	2	15	5	12	−0.18	1.0
blue – magenta	4	24	16	12	−0.43	$\text{2}.\text{3}\times {\text{10}}^{-\text{2}}$

Yes and No indicate whether a gene is significantly differentially expressed between FECD cases and controls or not by meta-analysis. For each module pair, the difference of proportions of differentially expressed genes between RE+ and RE- was calculated. Fisher’s exact test and McNermar’s test were used to compare the distributions of all the genes and only the shared genes, respectively. The nominal p-values were multiplied by seven to adjust for multiple testing.

**Fig 2:**
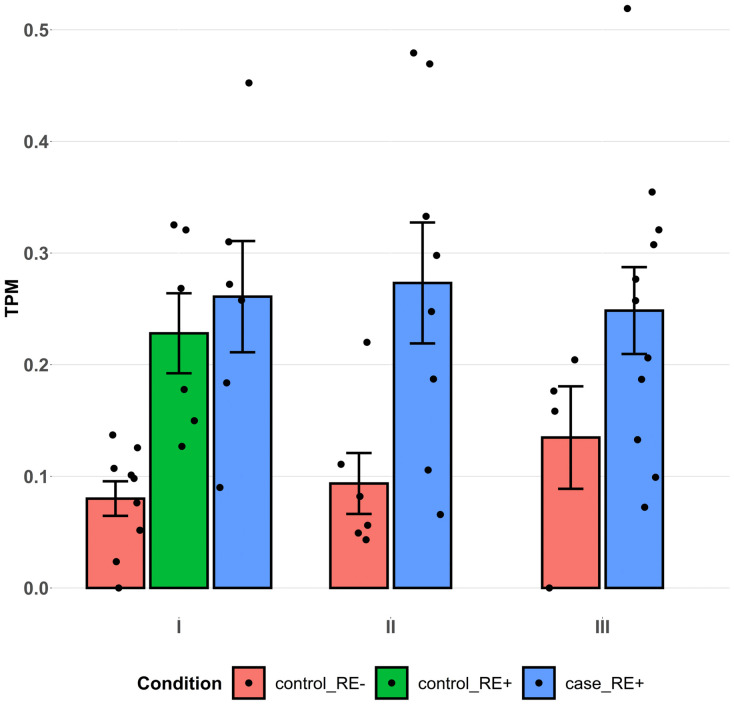
Differential expression of *RBM20* in the bulk RNA-seq datasets. Datasets I, II, and III refer to the three studies, I, II, and III, defined in Table 1. Each dot represents one sample. The y-axis indicated the expression levels of *RBM20* measured by transcripts per million (TPM). *RMB20* was significantly up-regulated in RE+ cases compared with in RE- controls by meta-analysis (FDR = 0.0046), and in RE+ controls compared with in RE- controls in dataset I (FDR = 0.0085).

There were a total of 2541 upstream regulator meeting the criterion of P<0.05 based on the differential expression analysis of RE+ cases vs. RE- controls, among which 1401 were expressed in the cornea endothelial tissue as detected in the bulk RNA-seq datasets (S2 Table). There were 7 regulators that were differentially expressed in RE+ cases but not in RE- cases, and with differentially expressed targets in the RE+ brown module (FDR < 0.05; Table 3). Among the 7 regulator genes, *RMB20* was also significantly up-regulated in RE+ controls compared with RE- controls, which was consistent with its up-regulation in RE+ cases (Fig 2).

**Table 3:** Differentially expressed upstream regulator genes with differentially expressed target genes in the brown module of Fuchs’ endothelial corneal dystrophy (FECD) co-expression network with the *TCF4* CTG18.1 expansion.

Gene	RE+ case vs. RE- control		RE- case vs. RE- control		RE+ control vs. RE- control		Targets
	Direction	FDR	Direction	FDR	LogFC	FDR	
*SOX9*	down	$\text{2}.\text{5}\times {\text{10}}^{-\text{5}}$	–	$\text{2}.\text{3}\times {\text{10}}^{-\text{1}}$	-0.95	$\text{5}.\text{2}\times {\text{10}}^{-\text{1}}$	*SULF2*
*NPM1*	down	$\text{9}.\text{2}\times {\text{10}}^{-\text{5}}$	down	$\text{7}.\text{9}\times {\text{10}}^{-\text{2}}$	-0.18	$\text{7}.\text{1}\times {\text{10}}^{-\text{1}}$	*NPM1*
*CAB39L*	up	$\text{8}.\text{1}\times {\text{10}}^{-\text{4}}$	up	$\text{3}.\text{3}\times {\text{10}}^{-\text{1}}$	0.37	$\text{4}.\text{1}\times {\text{10}}^{-\text{1}}$	*ATP5E*
*HSP90B1*	down	$\text{1}.\text{4}\times {\text{10}}^{-\text{3}}$	down	$\text{7}.\text{6}\times {\text{10}}^{-\text{2}}$	0.17	$\text{7}.\text{9}\times {\text{10}}^{-\text{1}}$	*RPS14, RPLP2*
***RBM20***	**up**	$4.6\times 10^{-3}$	up	$2.0\times 10^{-1}$	1.67	$8.5\times 10^{-3}$	***RPS8, NPM1, RPS12, RPS3A***
*NF1*	up	$\text{2}.\text{0}\times {\text{10}}^{-\text{2}}$	up	$\text{1}.\text{1}\times {\text{10}}^{-\text{1}}$	-0.09	$\text{9}.\text{0}\times {\text{10}}^{-\text{1}}$	*NPM1, RPS19*
*TCF12*	down	$\text{2}.\text{5}\times {\text{10}}^{-\text{2}}$	–	$\text{1}.\text{9}\times {\text{10}}^{-\text{1}}$	-0.57	$\text{1}.\text{1}\times {\text{10}}^{-\text{1}}$	*RPS3A*

RE+ and RE- refer to with and without the *TCF4* CTG18.1 expansion allele (≥40 repeats). FDR: false discovery rate; logFC: log_2_(fold change). The differential expression meta-analysis of RE+ case vs. RE- control and RE- case vs. RE- control was previously described [31]. In the Direction column, “up” and “down” indicate consistent differential expression directions among studies, whereas “–” indicates inconsistency. The RE+ control vs. RE- control comparison was based on 6 RE+ controls and 9 RE- controls in dataset I (GEO: GSE142538). The Targets column includes the differentially expressed targets of the upstream regulators that are in the FECD RE+ brown module.

**Fig 3:**
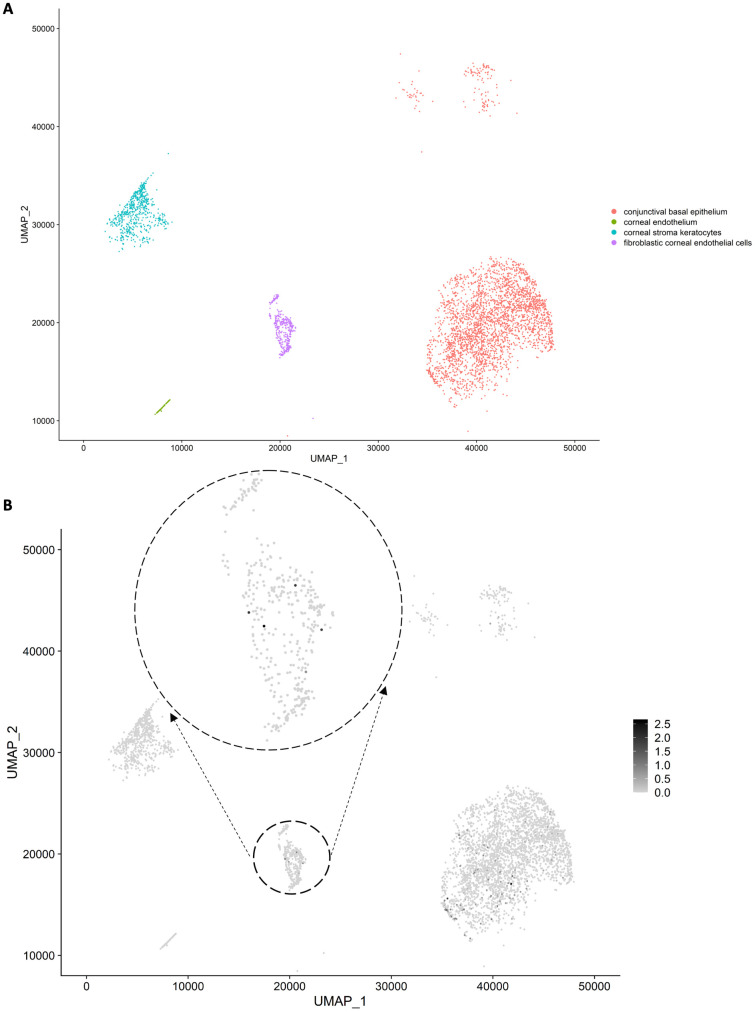
Expression of RBM20 in adult human cornea and adjacent conjunctiva single cells. (A) Single cell clusters of corneal endothelium, fibroblastic corneal endothelial cells, conjunctival basal epithelium, and corneal stroma keratocytes visualized by the Uniform Manifold Approximation and Projection (UMAP) plot. The single-cell RNA sequencing dataset (GEO:GSE155683) was generated using 4 adult cornea-conjunctival tissues [47]. The gene expression matrix and annotations of cell types were downloaded from http://retinalstemcellresearch.co.uk/CorneaCellAtlas/. (B) The cluster of fibroblastic corneal endothelial cells was amplified with the expression levels of *RBM20* highlighted. *RMB20* was not expressed in the corneal endothelium cell cluster; however, it was expressed in part of cells in the fibroblastic corneal endothelial cell cluster.

As the expression levels of *RBM20* were overall low in the bulk RNA-seq data (Fig 2), we hypothesized its expression was cell-type specific in the corneal endothelial tissues. Indeed in a single cell atlas of human cornea based on tissues from 4 adult doners, for whom neither the FECD phenotype nor CTG18.1 repeat expansion genotype were documented [47], *RBM20* was not expressed in the corneal endothelium cell cluster; however, it was expressed in part of cells in the fibroblastic corneal endothelial cell cluster (Fig 3). This observation corroborates the involvement of *RBM20* in FECD given the activation of fibrosis pathways in FECD [30,33].

### Motif and alternative splicing analyses of RMB20 targets

We confirmed the involvement of RMB20 in both RE+ cases and RE+ controls by examining the enrichment of its motif UCUU in the differential splicing events. There was an enrichment of this motif in the SE events in the upstream intronic region nearby an exon leading to down-regulated inclusion of the alternative exon; this phenomenon was consistent in each dataset for the AS analysis of RE+ cases vs. RE- controls (S1A Fig) and RE+ cases vs. RE- cases (S1B Fig). Moreover, it was observed in the comparisons of RE+ controls vs. RE- controls (S1D Fig) and RE+ cases vs RE+ controls (S1E Fig). In contrast it was not observed in the SE events of RE- cases vs. RE- controls (S1C Fig). There was no consistent enrichment of RBM20 motif in the MXE events (S2 Fig).

**Fig 4:**
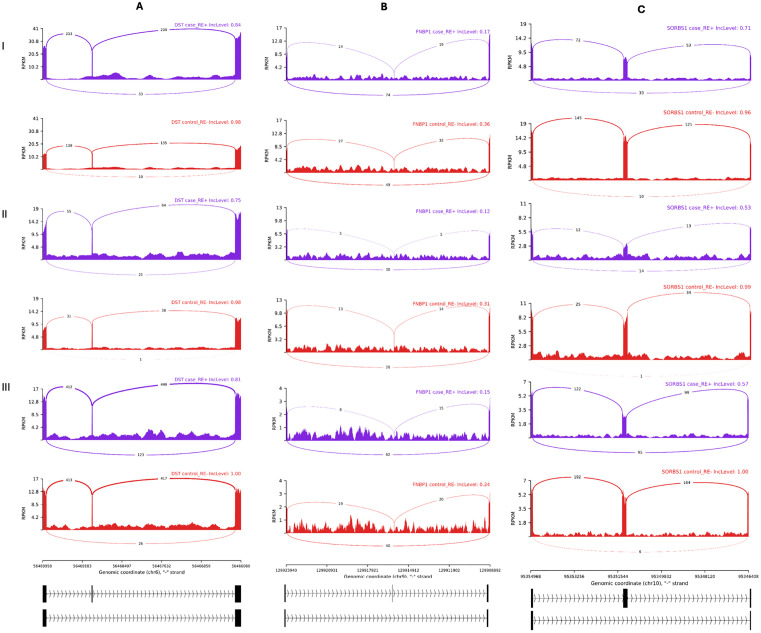
Three differential alternative splicing events in Fuchs’ endothelial corneal dystrophy patients with the *TCF4* CTG18.1 expansion compared with controls without the expansion. The Sashimi plots depicted the three skipped exon events, as described in Table 4, observed in all the 3 individual studies of RE+ cases vs RE- controls. A, B, and C refer to the three genes, *DST*, *FNBP1*, and *SORBS1*, respectively. I, II, and III refer to the three datasets, I, II, and III, defined in Table 1. The average inclusion level of each group (the percentages of the transcripts with the alternative exon) was labelled at the upper right corner. The y-axis indicated the average read density measured by reads per kilobase per million mapped reads (RPKM), and the average number of junction-spanning reads were labeled on the curves. Genome Reference Consortium Human Build 38 coordinates and relevant exons were drawn below.

**Fig 5:**
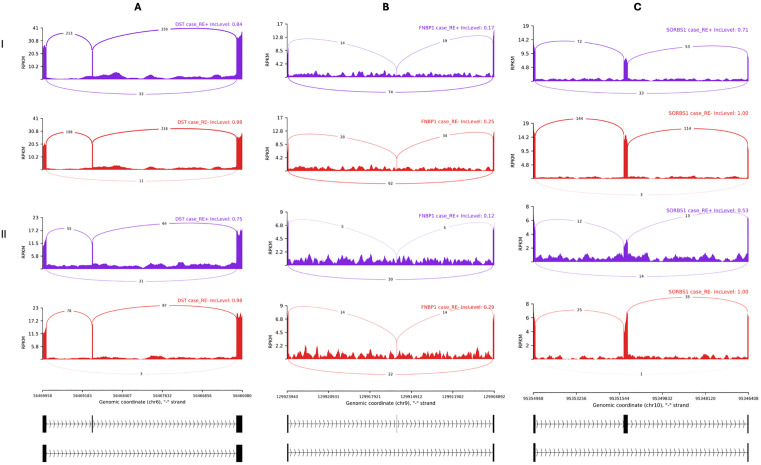
Three differential alternative splicing events in Fuchs’ endothelial corneal dystrophy patients with the *TCF4* CTG18.1 expansion compared with patients without the expansion. The Sashimi plots depicted the three skipped exon events, as described in Table 4, in the two studies of RE+ cases vs RE- cases. A, B, and C refer to the three genes, *DST*, *FNBP1*, and *SORBS1*, respectively. I and II refer to the two datasets, I and II, defined in Table 1. The average inclusion level of each group (the percentages of the transcripts with the alternative exon) was labelled at the upper right corner. The y-axis indicated the average read density measured by reads per kilobase per million mapped reads (RPKM), and the average number of junction-spanning reads were labeled on the curves. Genome Reference Consortium Human Build 38 coordinates and relevant exons were drawn at the bottom.

**Fig 6:**
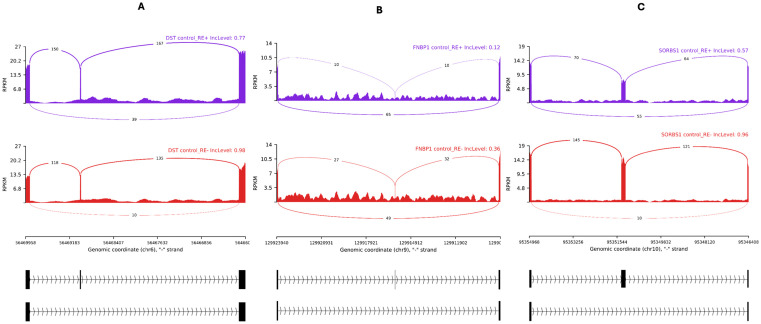
Three differential alternative splicing events in controls with the *TCF4* CTG18.1 expansion compared with controls without the expansion. The Sashimi plots depicted the three skipped exon events, as described in Table 4, comparing RE+ controls with RE- controls in dataset I defined in Table 1. A, B, and C refer to the three genes, *DST*, *FNBP1*, and *SORBS1*, respectively. The average inclusion level of each group (the percentages of the transcripts with the alternative exon) was labelled at the upper right corner. The y-axis indicated the average read density measured by reads per kilobase per million mapped reads (RPKM), and the average number of junction-spanning reads were labeled on the curves. Genome Reference Consortium Human Build 38 coordinates and relevant exons were drawn at the bottom.

The results of motif enrichment analysis suggested that increasing *RBM20* expression in RE+ samples would repress the inclusion of alternative exons. We next examined the SE events in 45 genes documented to be RBM20 targets in the literature (S1 Table) [42,44]. There were three events in *DST*, *FNBP1* and *SORBS1*, respectively, with downregulated inclusion levels of the target exons in RE+ cases compared with in RE- controls in each of the three studies (Table 4; Fig 4). The same phenomena were also observed when comparing RE+ cases with RE- cases (Fig 5). More intriguingly, the inclusion levels of the target exons were downregulated in RE+ controls, too (Fig 6). In contrast, these AS events were not observed comparing RE- cases with RE- controls.

**Table 4:** Events of consistently skipped exons in three studies of Fuchs’ endothelial corneal dystrophy (FECD) cases with the *TCF4* CTG18.1 expansion compared with controls without the *TCF4* CTG18.1 expansion.

Gene	Chromosome	Strand	Target exon	Upstream exon	Downstream exon	Exon inclusion levels
*DST*	6	–	56,468,982 − 56,468,999	56,466,078 − 56,466,195	56,469,883 − 56,469,957	down
*FNBP1*	9	–	129,915,966 − 129,915,980	129,908,890 − 129,908,999	129,923,844 − 129,923,939	down
*SORBS1*	10	–	95,351,209 − 95,351,376	95,346,406 − 95,346,452	95,354,882 - 953,54,967	down

A differential splicing event is defined by meeting four criteria: (1) false discovery rate < 0.05; (2) change of inclusion level > 5%; (3) the difference of average inclusion or skipping read counts between the two conditions > 5; and (4) the average inclusion read counts > 5. The three events reported here met the criteria in each of the three studies, I, II, and III, defined in Table 1. The coordinates correspond to the Genome Reference Consortium Human Build 38.

**Fig 7:**
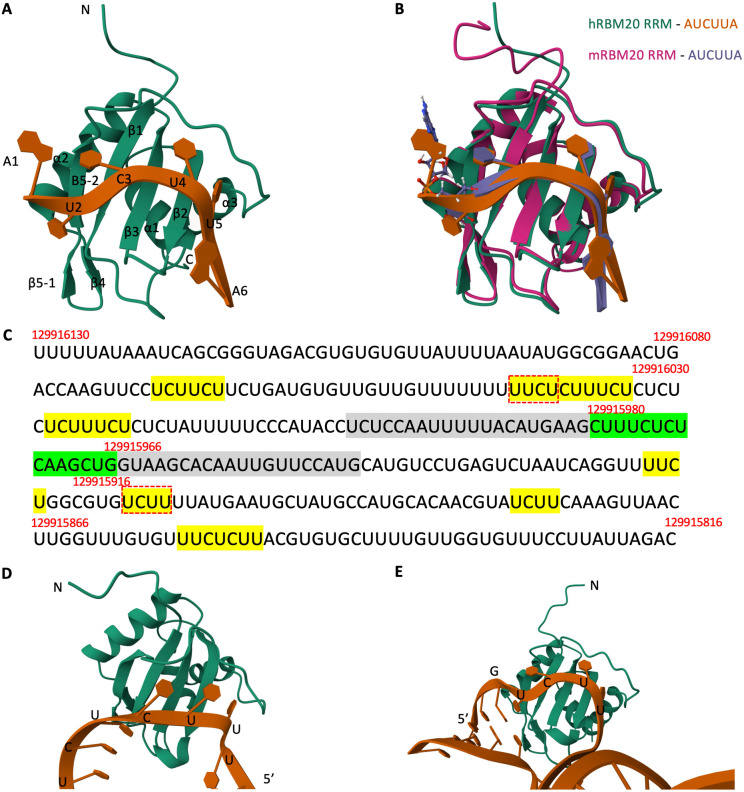
RBM20 RNA recognition motif (RRM)-RNA interaction predicted by AlphaFold 3. (A): Predicated structure of human RBM20 RRM domain (green) in complex with AUCUUA RNA (brown). The terminals, protein secondary structures and the nucleotides were annotated. The numbers after the nucleotides represented their positions in the fragments. (B): A comparison of the predicted structure in (A) with structure by NMR spectroscopy in mouse—RBM20 RRM domain (pink) and AUCUUA RNA (indigo). The mouse structure was downloaded from Protein Data Bank (PDB) with accession ID 6SO9 and superposed with the predicted structure in (A). The lowest energy structure model of 6SO9 with the lowest root mean square deviation (RMSD)—model 1 and RMSD = 1.54 Å—was presented. The two structures were superposed with the protein chains. (C): The RNA sequence (5’ → 3’) at chr9: 129,915,816−129,916,130 (hg38), containing the target exon (chr9: 129,915,966 − 129,915,980; green) of the skipped exon event at *FNBP1* and 150 nucleotides flanking intronic regions at both sides. The corresponding 1-based genome coordinates were labeled above the sequence. The flanking ±20 nucleotides were labeled in grey. The intronic UCUU and UUCU sequences were labeled in yellow. The motifs predicted to be bound by RRM, as in (D) and (E), were underlined with red boxes. AlphaFold 3 predicted the interactions between human RBM20 and fragments of *FNBP1* at 5’ intronic region (D) and 3’ intronic region (E) of the target exon, respectively. Only the interaction regions of the RNA fragments were presented.

We used AlphaFold 3 to predict the interaction structure of the human RBM20 RRM domain and the AUCUUA RNA oligo (Fig 7A). The predicted structure was similar to that of mouse RBM20 determined by nuclear magnetic resonance spectroscopy [41] (Fig 7B), which validated the predicated model by AlphaFold 3. Next we used it to investigate the interaction between the RBM20 RRM domain and the intronic flanking regions of the *FNBP1* exon 11 (Fig 7C), of which the inclusion levels were down-regulated in both RE+ cases and RE+ controls. For the 5’ intronic region, the domain was predicated to interact with the UUCU region at chr9:129,916,037−129,916,040 (Fig 7D); for the 3’ intronic region, the domain was predicated to interact with the UCUU region at chr9: 129,915,909−129,915,912 (Fig 7E). Predication of the interaction was also performed between the RBM20 RRM domain and the intronic flanking regions of the *SORBS1* exon 25, of which the inclusion levels were downregulated in both RE+ cases and RE+ controls (S3 Fig). The RRM domain was predicated to interact with the 3’ UUCU region at chr10: 95,351,113−95,351,116. The structures of both interactions were similar to that of the RRM domain and the AUCUUA RNA oligo. There was no predicted interaction between the domain and the UCUU/UUCU region at the flanking region of *DST* exon 98 (S4 Fig). A caveat to interpret the result is that the predicted model might not be the correct one, and it did not exclude the possibility that RBM20 regulates the AS of *DST* transcript by binding to other regions.

## Discussion

The RE+ and RE- late-onset FECD transcriptomic profiles showed high similarity in terms of differential expression compared to control profiles [33], which might be partially due to the ascertainment bias of limiting to the advanced stage FECD patients. To identify to the differences of molecular mechanisms between RE+ and RE- FECD we integrated differential expression analysis with co-expression analysis. Gene co-expression analysis clusters genes with similar expression patterns into modules that imply distinct biological processes and functions [49]. Although FECD RE+ and RE- shared module pairs with overlapped gene constitutions, the distributions of DEGs in a module pair were not necessarily consistent. Indeed, only between the brown-brown module pairs the proportions of DEGs were higher in RE+ than in RE-. We hypothesize that the transcriptomic (dys)regulation of the brown module genes is RE+ FECD specific. The translational initiation process was enriched with genes in the brown module pairs; however, when performing pathway analysis based on genes with different expression levels, it was only enriched in RE+ but not in RE- profiles [33]. It suggested a possible change in the ribosomal biogenesis in RE+ different from RE-. In the literature it was shown perturbation of TCF4 could disrupt ribosomal biogenesis [56,57]. We speculate that aberrant TCF4 transcripts in the RE+ patients could lead to down-regulation of genes encoding ribosomal constitution-related proteins.

In this study we identified RBM20, a splicing regulator, as an RE+ specific factor. Its expression levels were upregulated in both RE+ cases and RE+ controls (Fig 2). Consistently, its motif was enriched in the SE events of RE+ subjects compared with RE- subjects. It is of interest that the enrichment was also observed in the comparison of RE+ cases vs. RE+ controls (S1E Fig), which suggested a cumulative effect of RBM20 over time. This observation is in line with the increased expression of *RBM20* in the fibroblastic corneal endothelial cells (Fig 3B) and the dose-dependent effects of RMB20 on splicing [43]. However, it is unclear how RBM20 and the CTG18.1 trinucleotide repeat expansion are connected in the pathogenicity of FECD. By literature review, one hypothetical model is that the Wnt signaling pathway is interrupted. Experiments showed *RBM20* is a target of transcriptional factors TCF4 and ZEB1 [58,59]. Note that *ZEB1* was significantly downregulated in RE+ cases ($\text{FDR}\;=\;1.98\times 10^{-6}$) but not in RE- cases (FDR = 0.52) in the differential expression meta-analysis [33]. Missense mutations in *ZEB1* were reported to cause FECD [15]. It was shown the TCF4/ β-catenin complex binds to the promoter of *ZEB1* and activates its expression [60]; in cancer TCF4 and ZEB1 reciprocally modulate each other’s transcriptional activity: ZEB1 enhances TCF4/β-catenin-mediated transcription and Wnt signaling switches ZEB1 from a repressor into an activator [61].

The pathogenic mechanisms of CTG18.1 repeat expansion in FECD remain to be elucidated, and multiple hypotheses have been proposed, including *TCF4* dysregulation and toxicity of the *TCF4* transcript with the expanded repeats (for a review, see, e.g., reference [29]). The Wnt signaling pathway hypothesis proposed in the last paragraph suggests that the dysregulation of *TCF4* in RE+ cases may play a critical role for the upregulation of *RBM20* expression. Studies in corneal endothelium and primary patient-derived corneal endothelial cells identified an upregulated inclusion of an additional exon region of *TCF4*, referred to as E174, in RE+ cases [62,63]. The direct connection of this region to its 5′ exon 2 causes the original coding sequence to be included in the 5′ untranslated region. The translated product therefore loses 24 amino acid residues (MHHQQRMAALGTDKELSDLLDFSA) at the N-terminal of transactivation domain AD1 [64]. The LDFS motif inside this sequence was shown to be necessary for the AD1-mediated transactivation through recruitment of histone acetyltransferases [65,66]. In sum, we speculate the dysregulation of *TCF4*—rather than the repeat-associated toxicity—may be responsible for the enhanced *RBM20* expression in RE+ FECD.

It is of interest that in the scRNA-seq dataset *RBM20* was not expressed in the corneal endothelium cell cluster but expressed in part of cells in the fibroblastic corneal endothelial cell cluster. It indicates *RBM20* is associated with cellular transition in corneal endothelium. Endothelial-mesenchymal transition (EndMT) is recognized as a major contributor to fibrotic disease in multiple organs such as heart, kidney, lung, liver, etc. [67]. Recently its involvement in FECD was noted with various causes and mechanisms [68–70], which is not a surprise since FECD is a fibrotic disease with excessive accumulation of extracellular matrix (ECM) presented as guttae. In cancer ZEB1 is an epithelial-to-mesenchymal (EMT) transition activator, and the TCF4/ β-catenin complex induces ZEB1 to regulate tumor invasiveness. We speculate ZEB1 is also an EndMT activator, and thus the *RMB20* expression pattern could fit in the Wnt signaling pathway proposed.

There were SE events in three genes—*DST*, *FNBP1* and *SORBS1*—consistently identified in RE+ subjects (Figs 4−6) out of 45 genes documented to be RBM20 targets in the literature. However, it is known that in the RE+ subjects the expanded CUG repeat RNA transcripts can bind and sequester RNA splicing factors such as MBNL1 and MBNL2 and lead to widespread splicing dysregulation. Therefore, the mis-splicing effect of RBM20 could be confounded. It was previously shown that knockdown of *MBNL1* increased the inclusion levels of *FNBP1* exons in human monocytes [71]. According to the primers’ sequences, the relevant exons included the skipped exon at chr9:129,915,966−129,915,980 that we discovered herein. In another study using mouse C2C12 cells, *Mbnl1* and *Mbnl2* knockdowns did not alter *FNBP1* splicing [72]. In particular, the target exon studied (chr2:30,934,894−30,934,908 in mouse reference genome mm39) corresponded to the human copy we discussed herein. Therefore, the decreased inclusion levels of this *FNBP1* exon in RE+ suggested that this SE event be more likely caused by RBM20 than by MBNL1. In contrast, it was shown knockout of *MBNL1* could result in reduced inclusion of the *SORBS1* exon at chr10:95,351,209−95,351,376 [73]. Thus, this SE event could be regulated by either or both RBM20 and MBNL1. In sum, MBNL1/2 and RBM20 could function in independent, synergetic, or antagonistic ways. We could not find informative materials on DST in this regard in the literature. All the three genes—*DST*, *FNBP1* and *SORBS1*—were related to biological processes of cytoskeleton and extracellular matrix organization, pathways enriched in late-stage FECD, though their exact function in FECD is unclear [33]. We previously reported the splicing changes and perturbation of ECM genes seen in RE+ patients started long before symptoms were observable, as in RE+ controls [30], and the current results are in line with it. However, we were restricted to the toxic CUG repeat RNA theory when interpreting the results. With the recent discoveries supporting the *TCF4* dysregulation model [62,63], we suspect both models account for the phenomenon.

We acknowledge that the results of the current bioinformatic analyses require additional experimental validation. In this study we only examined AS in the 45 known targets based on cardiomyopathy-related studies, though there was a consistent enrichment of RBM20 motif in RE+ subjects in all potential SE events. As the expression level of *RBM20* in human corneal endothelial cells is low and it only starts to increase in those acquiring a fibroblast cell phenotype, cross-linking immunoprecipitation (CLIP) sequencing experiments in FECD-patient derived corneal endothelial cell lines are warranted to investigate RBM20’s target in corneal endothelial cells. For experiments based on clinical samples, considering the small amount of human corneal endothelial cells, sCLIP seems to be a promising approach to employ—it uses a linear *in vitro* transcription amplification reaction, avoiding RNA-ligation on low input material and omitting size-selection of cDNAs [74].

The RNA splicing process was negatively enriched in both RE+ and RE- FECD, and there were widespread splicing changes in both types [33]. The current study is based on short-read sequencing; in addition, the differential expression analyses of transcripts and differential splicing analyses rely on the documented annotations. Considering the complexity of AS and the possible novel splicing events in the disease tissues, long-read sequencing is necessary to depict the transcriptomic profile of FECD at the transcript level.

## Supporting information

S1 Fig RBM20 motif enrichment analysis in the skipped exon (SE) alternative splicing events. Software rMAPS calculates the motif scores at the regions with differential SE events (FDR < 0.05 and change of inclusion level > 5%) identified by rMATS by determining the occurrence densities of RBM20 motif (UCUU/ TCTT) in them. The solid lines are the motif scores with up-regulated (red), down-regulated (blue) and non-differential background (black) exon inclusions. The dashed lines are the -log(P-value) by Wilcoxon’s rank sum test comparing the motif scores of up-regulated (red) or down-regulated (blue) events with the background. (A): RE+ cases vs. RE- controls; (B): RE+ cases vs. RE- cases; (C): RE- cases vs. RE- controls; (D): RE+ controls vs. RE- controls; (E): RE+ cases vs RE+ controls.
(PDF)

S2 Fig RBM20 motif enrichment analysis in the mutually exclusive exon (MXE) alternative splicing events. Software rMAPS calculates the motif scores at the regions with differential MXE events (FDR < 0.05 and change of inclusion level > 5%) identified by rMATS by determining the occurrence densities of RBM20 motif (UCUU/ TCTT) in them. The solid lines are the motif scores with up-regulated (red), down-regulated (blue) and non-differential background (black) exon inclusions. The dashed lines are the -log(P-value) by Wilcoxon’s rank sum test comparing the motif scores of up-regulated (red) or down-regulated (blue) events with the background. (A): RE+ cases vs. RE- controls; (B): RE+ cases vs. RE- cases; (C): RE- cases vs. RE- controls; (D): RE+ controls vs. RE- controls; (E): RE+ cases vs RE+ controls.
(PDF)

S3 Fig RBM20 RNA recognition motif (RRM)-*SORBS1* RNA interaction predicted by AlphaFold 3. (A): The RNA sequence (5’ → 3’) at chr10: 95,351,059−95,351,526 (hg38), containing the target exon (chr10: 95,351,209−95,351,376; green) of the skipped exon events at *SORBS1* and 150 nucleotides flanking intronic regions at both sides. The corresponding 1-based genome coordinates were labeled above the sequence. The flanking ±20 nucleotides were labeled in grey. The intronic UCUU and UUCU sequences were labeled in yellow. The motif predicted to be bound by RRM, as in (B), was underlined with red boxes. AlphaFold 3 predicted the interactions between human RBM20 and fragment of *SORBS1* at 3’ intronic region (B) of the target exon. Only the interaction regions of the RNA fragments were presented.
(PDF)

S4 Fig RBM20 RNA recognition motif (RRM)-*DST* RNA interaction predicted by AlphaFold 3. (A): The RNA sequence (5’ → 3’) at chr6: 56,468,832−56,469,149 (hg38), containing the target exon (chr6: 56,468,982–56,468,999; green) of the skipped exon events at *DST* and 150 nucleotides flanking intronic regions at both sides. The corresponding 1-based genome coordinates were labeled above the sequence. The flanking ±20 nucleotides were labeled in grey. The intronic UCUU and UUCU sequences were labeled in yellow. (B): We tried to use AlphaFold 3 to predict the binding sites of human RBM20 on the flanking intronic region of the *DST* transcript with 6 RNA fragments: (1) 150 bp 5’ flanking intronic region, (2) 100 bp 5’ flanking region, (3) the 5’ intronic region containing all the UCUU/UUCU motifs within the 150 bp range as well as 4 extra nucleotides at both ends, (4) 150 bp 3’ flanking intronic region, (5) 100 bp 3’ flanking region, (6) the 3’ intronic region containing all the UCUU/UUCU motifs within the 150 bp range as well as 4 extra nucleotides at both ends. None of the interactions predicted UCUU/UUCU motif as the binding motif, and the best model was presented in (B) by using fragment (3). The motif predicted to be bound by RRM in (B) was underlined with red boxes in (A). Only the interaction regions of the RNA fragments were presented.
(PDF)

S1 Table A list of RBM20 target genes in the literature. (XLSX)

S2 Table The upstream regulators inferred based on the differential expression meta-analysis of Fuchs’ endothelial corneal dystrophy cases with the *TCF4* CTG18.1 expansion compared with controls without the *TCF4* CTG18.1 expansion. (XLSX)
