# Supplementary figures and images for: Transcriptomic analysis implicates the involvement of RBM20 in Fuchs’ endothelial corneal dystrophy with TCF4 repeat expansion
Source: PLoS One. 2025 Sep 17;20(9):e0332512. doi: 10.1371/journal.pone.0332512 (PMC12443318; doi:10.1371/journal.pone.0332512)

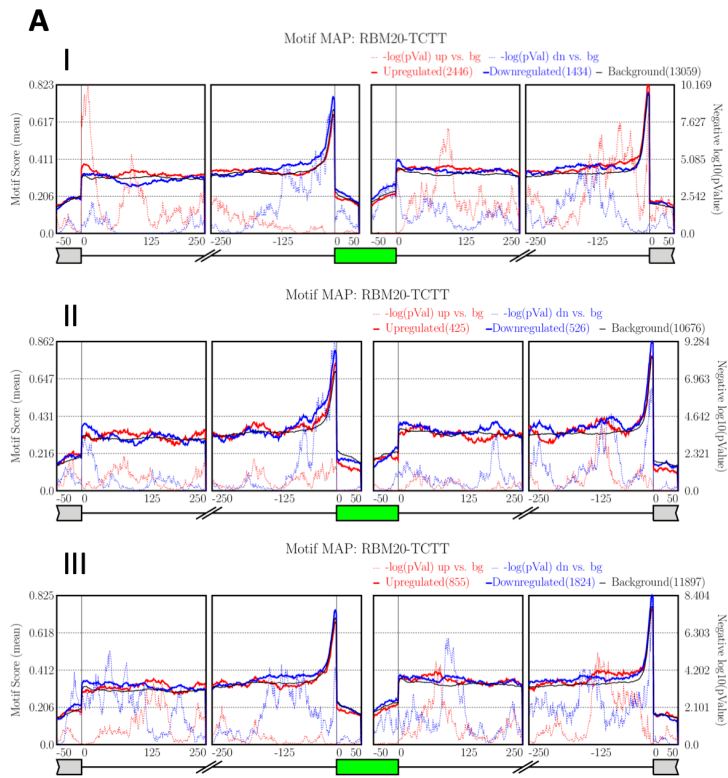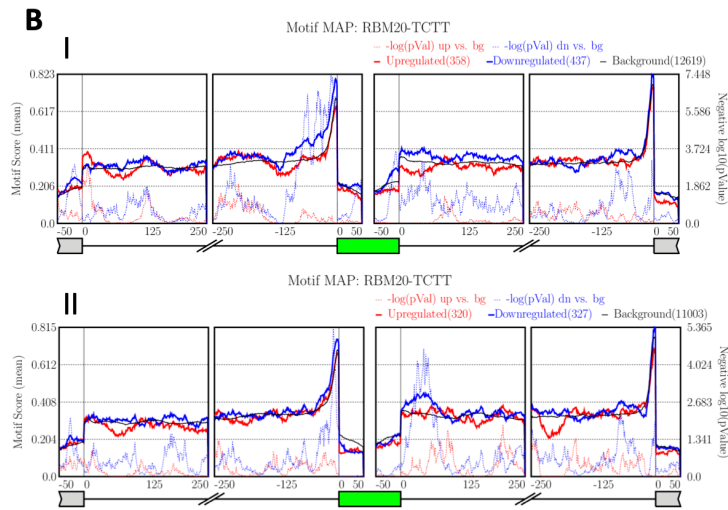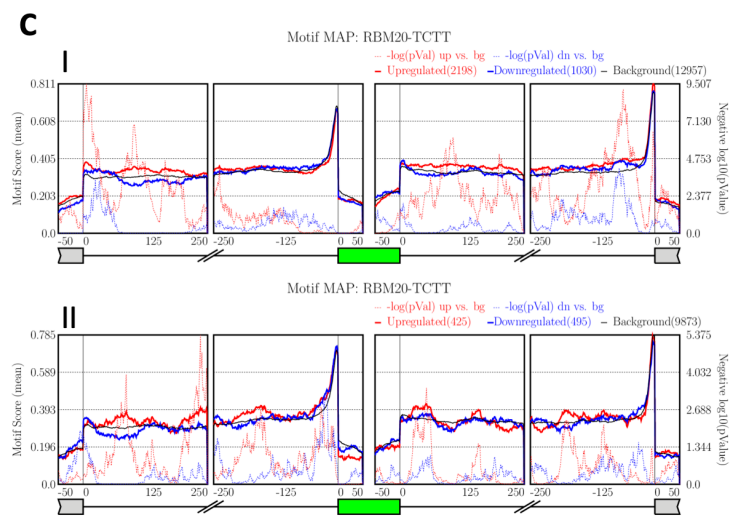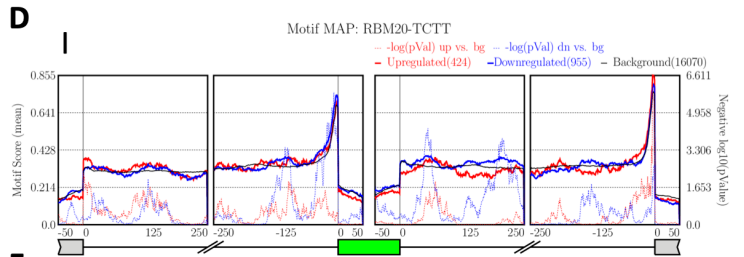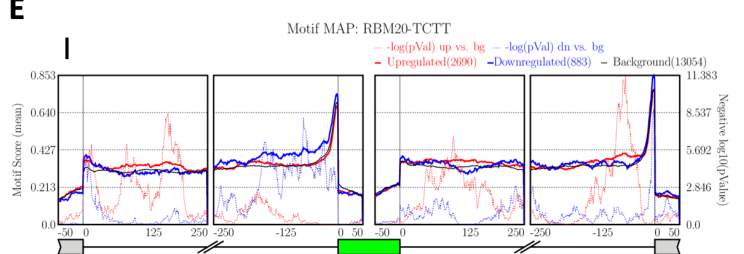

Supplement: S1 Fig — Software rMAPS calculates the motif scores at the regions with differential SE events (FDR < 0.05 and change of inclusion level > 5%) identified by rMATS by determining the occurrence densities of RBM20 motif (UCUU/ TCTT) in them. The solid lines are the motif scores with up-regulated (red), down-regulated (blue) and non-differential background (black) exon inclusions. The dashed lines are the -log(P-value) by Wilcoxon’s rank sum test comparing the motif scores of up-regulated (red) or down-regulated (blue) events with the background. (A): RE+ cases vs. RE- controls; (B): RE+ cases vs. RE- cases; (C): RE- cases vs. RE- controls; (D): RE+ controls vs. RE- controls; (E): RE+ cases vs RE+ controls. (PDF) [file pone.0332512.s001.pdf]

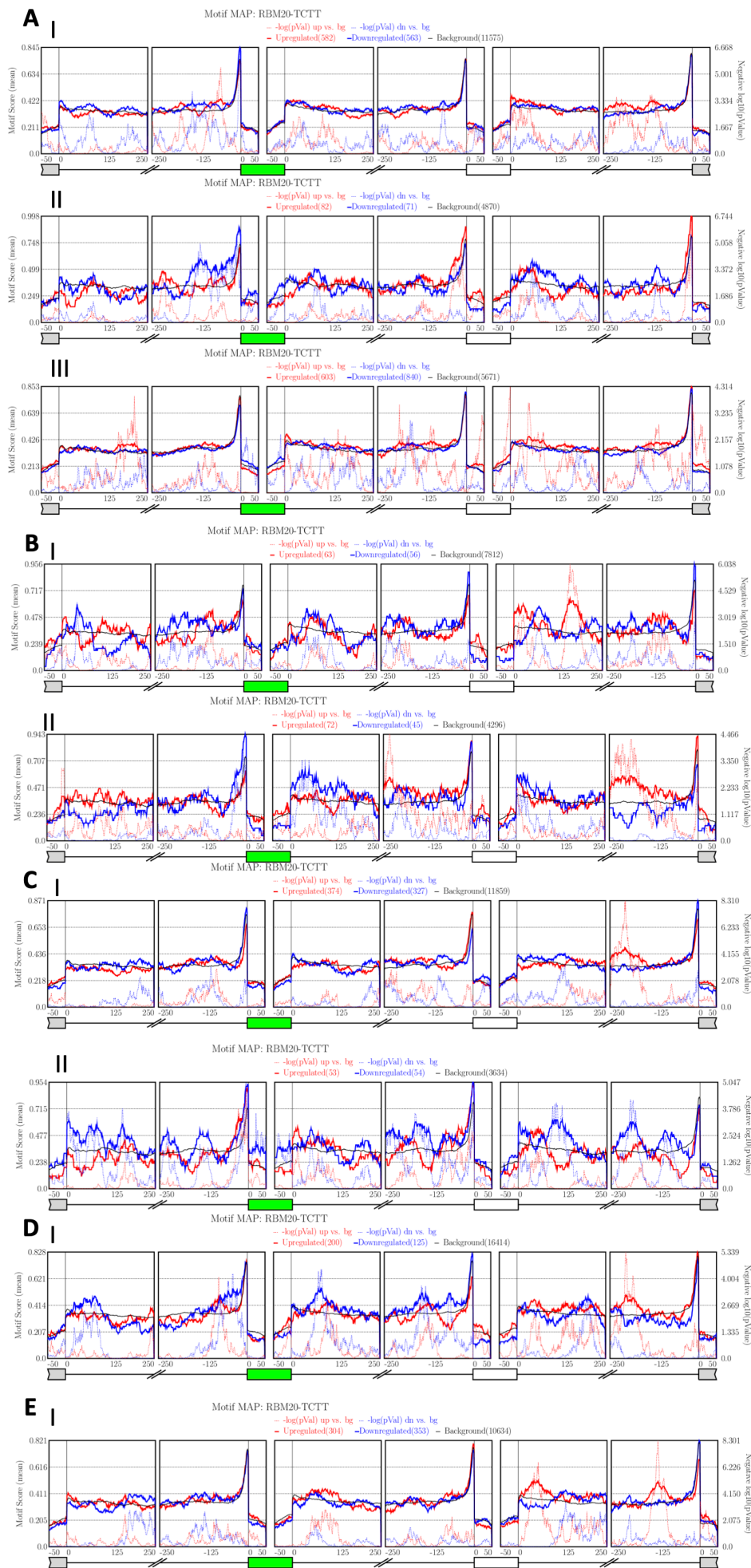

Supplement: S2 Fig — Software rMAPS calculates the motif scores at the regions with differential MXE events (FDR < 0.05 and change of inclusion level > 5%) identified by rMATS by determining the occurrence densities of RBM20 motif (UCUU/ TCTT) in them. The solid lines are the motif scores with up-regulated (red), down-regulated (blue) and non-differential background (black) exon inclusions. The dashed lines are the -log(P-value) by Wilcoxon’s rank sum test comparing the motif scores of up-regulated (red) or down-regulated (blue) events with the background. (A): RE+ cases vs. RE- controls; (B): RE+ cases vs. RE- cases; (C): RE- cases vs. RE- controls; (D): RE+ controls vs. RE- controls; (E): RE+ cases vs RE+ controls. (PDF) [file pone.0332512.s002.pdf]
