# Supplementary material for: Transcriptomic analysis implicates the involvement of RBM20 in Fuchs’ endothelial corneal dystrophy with TCF4 repeat expansion
Source: PLoS One. 2025 Sep 17;20(9):e0332512. doi: 10.1371/journal.pone.0332512 (PMC12443318; doi:10.1371/journal.pone.0332512)

A

95351526 95351476

UAGUUGUGGCUCUACAGCCGUUCUGACAGCUGAGUCUUAAAGCUUCUGCCCCA

95351426

UUCACCCAGUACCUCCUUCCCUAAUUACUUUUGUUGGCAGUUUUCUAAUAUGC

95351376

UGGAACAGAUGAUUCACUGCCCAUUUUAUGCAAUUUUAUGGCAGAAACUUUU

AGCGGACCAGAGACGACUUAAACGCGAGCAAGAAGAGGCUGAUAUUGCAGCUC

GACGCCACACAGGCGUCAUUCCGACGCACCAUCAGUUUAUCACUAAUGAGCGCU

95351209

UUGGGGACCUCCUCAAUAUAGACGAUACUGCAAAAAGGAAAUCUGGGUCAGAG

95351159

GUCUGUUUUGGUCACCUCAAUCUGCUGCUUGACCCAAAGCAAUAUUUGCCUA

95351109

UUUGCCUCCCCUCAGCAUUGUCCUCUGUAGGCCAGUUUAGUUCUCCAAACAGC

95351059

AUCUGUCAUCCUGGCCUCCCUUAGGCUUGCUUUGGCUUCUUGCGG

B

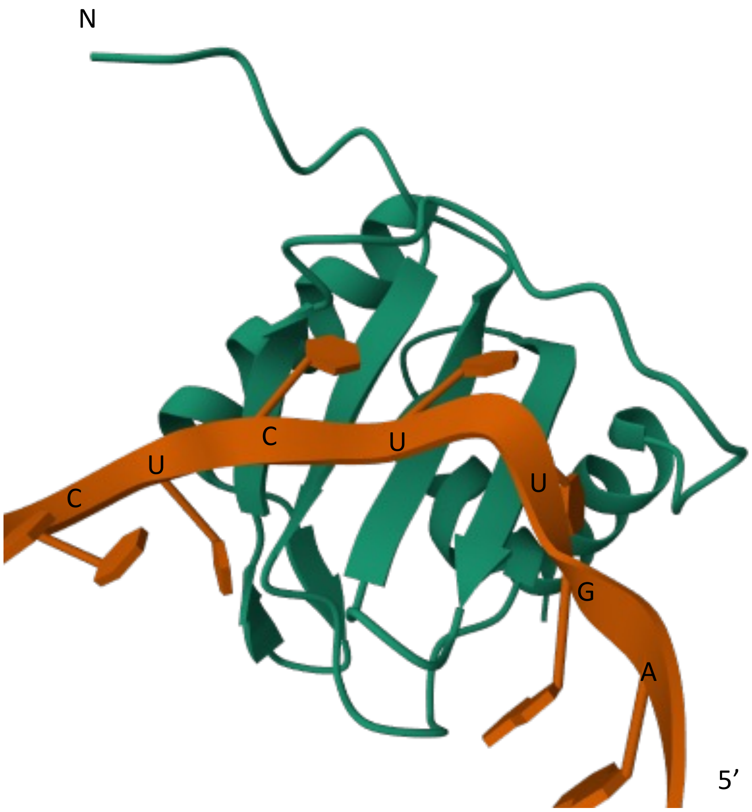

Supplement: S3 Fig — (A): The RNA sequence (5’ → 3’) at chr10: 95,351,059−95,351,526 (hg38), containing the target exon (chr10: 95,351,209−95,351,376; green) of the skipped exon events at SORBS1 and 150 nucleotides flanking intronic regions at both sides. The corresponding 1-based genome coordinates were labeled above the sequence. The flanking ±20 nucleotides were labeled in grey. The intronic UCUU and UUCU sequences were labeled in yellow. The motif predicted to be bound by RRM, as in (B), was underlined with red boxes. AlphaFold 3 predicted the interactions between human RBM20 and fragment of SORBS1 at 3’ intronic region (B) of the target exon. Only the interaction regions of the RNA fragments were presented. (PDF) [file pone.0332512.s003.pdf]
