# Supplementary material for: Transcriptomic analysis implicates the involvement of RBM20 in Fuchs’ endothelial corneal dystrophy with TCF4 repeat expansion
Source: PLoS One. 2025 Sep 17;20(9):e0332512. doi: 10.1371/journal.pone.0332512 (PMC12443318; doi:10.1371/journal.pone.0332512)

A

56469149 56469099
GAAAUUGGGACAACUGGGUGCCUGCACAUCUCUAAACUAGAUACUACAUCCAG

56469049
AAGCACAGAGUGUGAGUAUGUGUGUAUGUUCUAGAGUCAUACAGAAAGUUUC

56468999
CUGUUGAACUAAUUGUGUCUUUCUUCCAUUUUUCAUUUUUAUCAGUUCUUCC

56468982
UGGGAAAUCAGGUAUAAACCAGUGGAAUGUAUUCUCAAGUUCCUGAUGAUUU

56468932
UUUUUUAACUUUAAUUCAUAGUCAUUAAAGCUUGCCAUGGCCAAUCUUUCCC

56468882
AUGCUGUCUCCCUAGCUACUACAUUGUUGGUUGGCGUGGUGUUUCUGUAAAC

56468832
GCUAU

B

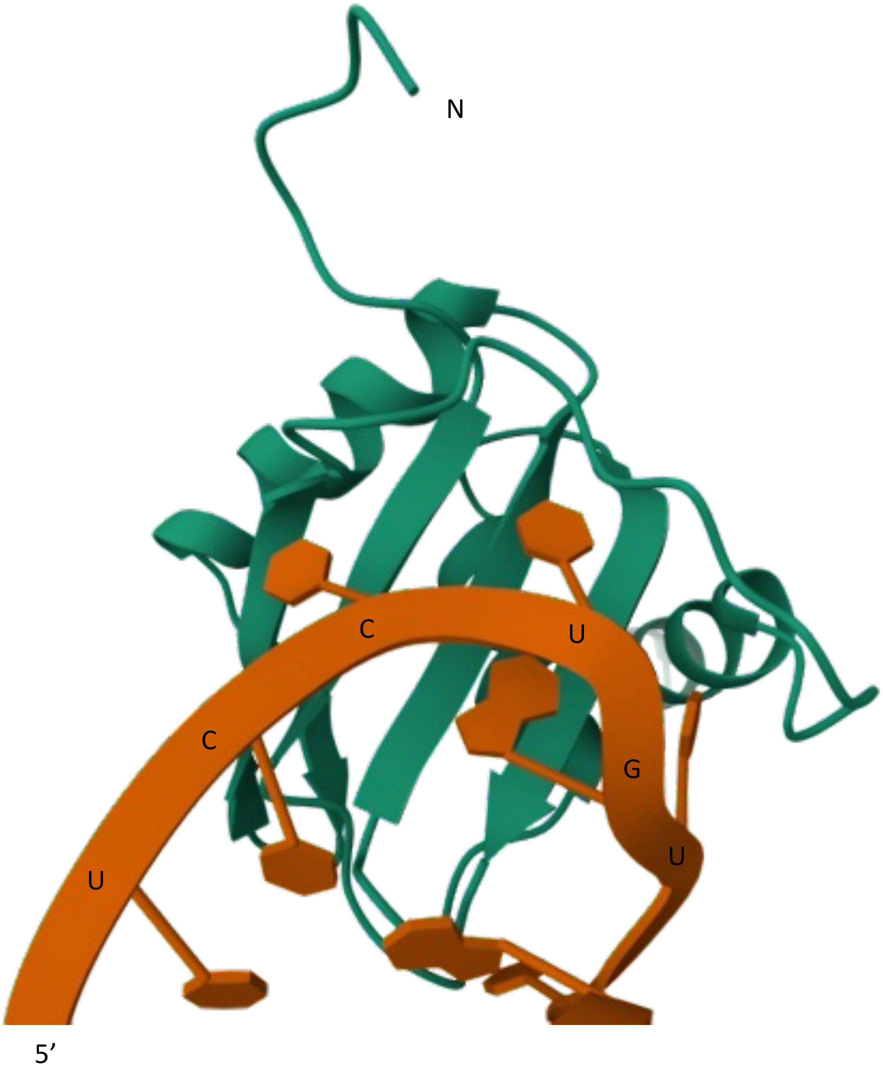

Supplement: S4 Fig — (A): The RNA sequence (5’ → 3’) at chr6: 56,468,832−56,469,149 (hg38), containing the target exon (chr6: 56,468,982–56,468,999; green) of the skipped exon events at DST and 150 nucleotides flanking intronic regions at both sides. The corresponding 1-based genome coordinates were labeled above the sequence. The flanking ±20 nucleotides were labeled in grey. The intronic UCUU and UUCU sequences were labeled in yellow. (B): We tried to use AlphaFold 3 to predict the binding sites of human RBM20 on the flanking intronic region of the DST transcript with 6 RNA fragments: (1) 150 bp 5’ flanking intronic region, (2) 100 bp 5’ flanking region, (3) the 5’ intronic region containing all the UCUU/UUCU motifs within the 150 bp range as well as 4 extra nucleotides at both ends, (4) 150 bp 3’ flanking intronic region, (5) 100 bp 3’ flanking region, (6) the 3’ intronic region containing all the UCUU/UUCU motifs within the 150 bp range as well as 4 extra nucleotides at both ends. None of the interactions predicted UCUU/UUCU motif as the binding motif, and the best model was presented in (B) by using fragment (3). The motif predicted to be bound by RRM in (B) was underlined with red boxes in (A). Only the interaction regions of the RNA fragments were presented. (PDF) [file pone.0332512.s004.pdf]
